# Image Sensor-Based Three-Dimensional Visible Light Positioning for Various Environments

**DOI:** 10.3390/s25154741

**Published:** 2025-08-01

**Authors:** Xiangyu Liu, Junqi Zhang, Song Song, Lei Guo

**Affiliations:** 1 The School of Information Science and Engineering, Shenyang Ligong University, Shenyang 110159, China; 15104178770@163.com; 2 The School of Communication and Information Engineering, Chongqing University of Posts and Telecommunications, Chongqing 400065, China; songsong@cqupt.edu.cn (S.S.); guolei@cqupt.edu.cn (L.G.); 3 Institute of Intelligent Communications and Network Security, Chongqing University of Posts and Telecommunications, Chongqing 400065, China

**Keywords:** visible light positioning, three-dimensional positioning, unscented particle filter algorithm

## Abstract

Research on image sensor (IS)-based visible light positioning systems has attracted widespread attention. However, when the receiver is tilted or under a single LED, the positioning system can only achieve two-dimensional (2D) positioning and requires the assistance of inertial measurement units (IMU). When the LED is not captured or decoding fails, the system’s positioning error increases further. Thus, we propose a novel three-dimensional (3D) visible light positioning system based on image sensors for various environments. Specifically, (1) we use IMU to obtain the receiver’s state and calculate the receiver’s 2D position. Then, we fit the height–size curve to calculate the receiver’s height, avoiding the coordinate iteration error in traditional 3D positioning methods. (2) When no LED or decoding fails, we propose a firefly-assisted unscented particle filter (FA-UPF) algorithm to predict the receiver’s position, achieving high-precision dynamic positioning. The experimental results show that the system positioning error under a single LED is within 10 cm, and the average positioning error through FA-UPF under no light source is 6.45 cm.

## 1. Introduction

**Figure 1:**
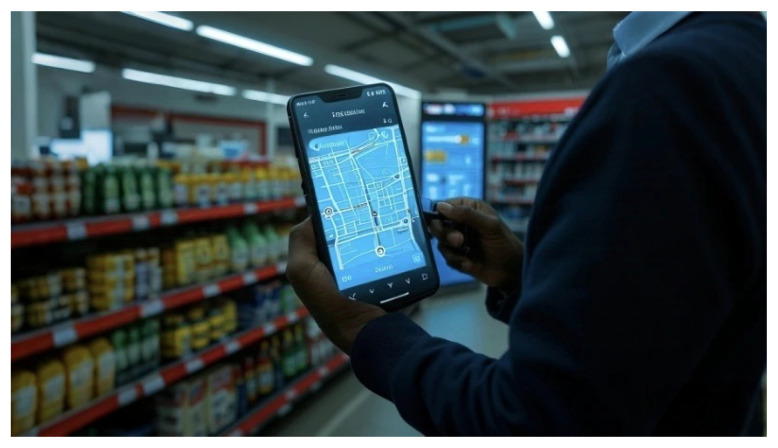
Application of VLP in supermarkets.

With the development of the internet and the continuous improvement of smart buildings, the high-precision location-based service (HPLBS) has become an indispensable part of urban modernization, and the increase in indoor activities has further increased the demand for indoor positioning [1]. Existing technologies such as wireless local area network (WLAN) [2], Bluetooth [3], infrared [4], ultrasonic (UT) [5], ultra-wideband (UWB) [6], and radio frequency (RF) [7] can be used to achieve indoor positioning. However, these technologies have some accuracy, cost, and robustness disadvantages. Visible light positioning (VLP) has the advantages of high accuracy and low deployment cost. It is not susceptible to electromagnetic interference and is used in various electromagnetic-sensitive positioning scenarios, such as hospitals and mines [8]. The visible light positioning system uses a light-emitting diode (LED) as the transmitter, which can provide positioning while considering the lighting function. Therefore, it has attracted widespread attention from scholars and has become a powerful alternative to traditional indoor positioning technologies [9]. Figure 1 shows the positioning of visible light in the house used in a supermarket, which makes it more efficient for people to find the goods.

Most research on visible light positioning systems based on image sensors uses multiple LED information for positioning. However, in complex indoor environments, the field of view of image sensors may be blocked by many obstacles, leading to positioning failure. In addition, this research also lacks height information of the receiver. Specifically as follows.

(1) Traditional visible light positioning algorithms require the image sensor to capture three or more complete LED images for high-precision positioning. When the light source layout is sparse or only one LED is decoded, the existing method uses positioning parameter information and inertial measurement unit (IMU) data to calculate the receiver position. However, the IMU has a drift error, seriously affecting the positioning accuracy.
(2) When the existing visible light positioning system realizes three-dimensional positioning, its algorithm simultaneously includes the receiver’s *X*, *Y*, and *Z* coordinates in the three-variable nonlinear equation system to solve the position. Slight parameter errors during the least squares iteration process will accumulate and amplify error in the final three-dimensional positioning result. In addition, solving nonlinear equations takes a long time.
(3) Some existing indoor visible light positioning systems use particle filter (PF) or unscented particle filter (UPF) combined with inertial measurement units (IMU) to achieve positioning. However, when the receiver moves, the LED image will be missed from the image sensor’s field of view (FoV). The particle filter algorithm cannot work correctly. In addition, the particle dilution problem in the later stage of the particle filter will decrease positioning accuracy [10].

To address the above issues, we propose a single LED three-dimensional visible light positioning system based on an image sensor. This system separates the height from the three-dimensional information and calculates it separately, avoiding the transmission and amplification of errors in solving nonlinear equations. Furthermore, when no LED is in the image sensor’s field of view (FoV) or decoding fails, we propose the firefly-assisted unscented particle filter (FA-UPF) algorithm to predict the receiver position, which improves the system’s positioning accuracy and robustness. The details are as follows:

(1) We propose a single LED three-dimensional positioning algorithm. It uses the geometric projection relationship of the image to list the similar triangles proportional equation for calculating the receiver’s *X* and *Y* coordinates, achieving high-precision two-dimensional positioning. Regarding height calculation, we establish a height–size database and fit the linear curve between the receiver’s height and the LED’s pixel width. After calculating the two-dimensional positioning, the pixel width of the LED is input into the linear curve to obtain the receiver’s height and achieve three-dimensional positioning. The positioning algorithm that separates the horizontal and vertical coordinates from the height avoids the accumulation and transmission of errors when solving nonlinear equation systems in traditional techniques.
(2) We propose the firefly-assisted unscented particle filter algorithm. It uses the firefly algorithm to optimize the particle distribution of the unscented particle filter, which prevents the particles from gathering at the local optimum after resampling and dramatically reduces the IMU’s error. When no LED exists in the image sensor’s field of view (FoV) or decoding fails, the algorithm can accurately predict the receiver’s position, solving the problem of dynamic positioning and increasing system robustness.
(3) Finally, we build an experimental platform to test the system’s performance. The experiment shows that in the 2 m × 2 m × 2.65 m positioning area, the average error of the system is 5.34 cm. The minimum positioning error is 0.76 cm, and the maximum positioning error is 15.89 cm. In addition, more than 70% of the test points have a positioning error within 10 cm. We also test the position prediction without LED; the results show that the system can achieve centimeter-level positioning accuracy.

In summary, this paper addresses key limitations in image sensor-based visible light positioning systems, including the inability to perform accurate 3D positioning with sparse LEDs and the degradation of prediction accuracy when LEDs are absent. To overcome these challenges, we propose a novel single-LED 3D positioning algorithm and a firefly-assisted unscented particle filter (FA-UPF) for dynamic positioning. The remainder of this paper is organized as follows: Section 2 reviews related work on visible light positioning and filtering methods. Section 3 introduces the system architecture and coordinate transformations. Section 4 details the proposed single LED positioning algorithm. Section 5 describes the FA-UPF algorithm for dynamic positioning. Section 6 presents the experimental setup and evaluation results, and Section 7 concludes the paper.

## 2. Related Work

In this section, we introduce visible light positioning and particle filter.

### 2.1. Visible Light Positioning

(1) Multiple LEDs case.

Liu et al. designed a real-time high-precision visible light positioning algorithm, which utilizes the trilateration algorithm to determine the receiver’s position when three LED beacons are within the field of view [11]. Li et al. proposed a three-LED positioning method that does not require encoding. The proposed algorithm arranges LEDs with different spacings in a straight line and determines the receiver’s position from the geometric relationship of the LEDs captured by the camera [12]. Zhu et al. proposed a positioning algorithm for circular LEDs, which calculates the camera position by utilizing the feature points of the LEDs and their arc-shaped geometric characteristics. The algorithm achieves a 10 cm positioning accuracy using only two circular LEDs [13]. Yazar et al. proposed a pulse optimization design method for LEDs as transmitters. The method quantifies positioning accuracy using the Cramér-Rao Lower Bound (CRLB) and investigates the maximization of positioning performance in both asynchronous and synchronous visible light positioning systems. It can reduce energy consumption by approximately 45% or improve positioning accuracy by 25% [14]. Huang et al. proposed an RSS-based VLP method that transforms time-domain signals to the frequency domain and jointly estimates receiver position, LED visibility indicators, and signal parameters to achieve robust positioning under limited FOV [15]. Cappelli et al. proposed a low-power Visible Light Localization (VLL) system using three modulated LEDs and a photodiode receiver, where embedded machine learning regressors on a microcontroller process light intensities to achieve energy-efficient indoor positioning with accuracy satisfying predefined error constraints [16].

(2) Single LED case.

Cheng et al. proposed an optical camera communication (OCC)-based visible light positioning system that improves positioning accuracy using a plane intersection method from the geometric features of LED projections. Experimental results show that the system achieves a 3D average positioning error of 5.5 cm under different heights and tilt angles, enabling high-precision positioning [17]. Mao et al. proposed a visible light positioning method using an improved Camshift algorithm and a 2D HSV histogram to enhance LED tracking accuracy and robustness. Experiments show a positioning accuracy of 0.95 cm, with 90% of errors below 1.79 cm, demonstrating high precision and real-time performance for dynamic positioning [18]. Hao et al. proposed an improved angle-of-arrival (AOA) method, in which the LED is at known locations as the transmitter, projecting the LED onto an image sensor with a camera acting as the receiver to capture the LED signals. The system can determine accurate position and orientation, achieving decimeter-level positioning [19]. Kim et al. proposed a tracking system based on a single LED and a single PD, which reduces the computational burden of the particle filter by applying a novel Bayesian method [20]. Chen et al. proposed a VLP positioning method that uses only a single LED light. This method measures the rotation angle by using an IMU and calibrates the IMU data with visual projection geometry, achieving sub-meter positioning accuracy [21]. Wang et al. proposed a single-LED visible light positioning system using a tilted rotatable photodetector (STRP) that accounts for first-order reflections, deriving the Cramer-Rao lower bound for optimal receiver angle selection and introducing an LS-assisted improved gray wolf optimization (LS-IGWO) algorithm to achieve centimeter-level positioning accuracy [22].

### 2.2. Filter Methods for Positioning

Liang et al. proposed the visible light positioning system with the tightly coupled extended Kalman filter (EKF) and IMU. The system fuses inertial measurements and visual information to achieve lightweight, real-time, and high-precision global positioning, maintaining stable performance even in sparse LED environments [23]. Guan et al. proposed an improved Camshift-Kalman algorithm, which combines the Camshift algorithm with a Kalman filter and innovatively introduces the Bhattacharyya coefficient. Experimental results demonstrate that the algorithm significantly enhances positioning accuracy, real-time performance, and robustness [24]. Liang et al. proposed a tightly coupled vision-inertial fusion method based on EKF, leveraging IMU and CMOS image sensors to enhance the system robustness [25]. Xie et al. proposed a novel visible light positioning method based on the Mean Shift (MS) algorithm and the Unscented Kalman Filter (UKF). In this method, the Unscented Kalman Filter enables the Mean Shift algorithm to track high-speed targets, improving positioning accuracy when the LED is obstructed [26]. Poompat et al. employed EKF to estimate joint position and orientation in an indoor visible light positioning system. The evaluation shows that 95% of the time, the position error is within 0.18 m and the orientation error is less than or equal to 12.5°, demonstrating the effectiveness of EKF in dynamic tracking [27].

## 3. System Overview

In this section, we introduce the composition of the visible light positioning system and the relationship between coordinate system transformations.

### 3.1. Visible Light Positioning System

**Figure 2:**
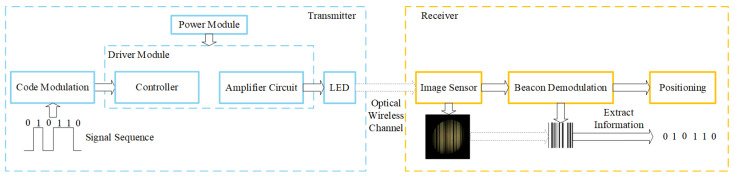
Framework of image sensor-based visible light positioning.

The framework of the visible light positioning system is shown in Figure 2. It can be divided into three parts: the transmitter, the channel, and the receiver [28]. First, the transmitter encodes and modulates the LED beacon data. Then, the driving circuit controls the LED to transmit the beacon information. The light signals carry the beacon information to propagate in the physical space through the visible light channel. The signal arrives at the photosensitive element of the image sensor at the receiver. The photosensitive element converts the received light signals into electrical signals. Then, the receiver performs signal processing, such as noise reduction, demodulation, decoding, etc., to restore the original signal. Finally, we calculate the receiver’s position according to the LED beacon information and the positioning algorithms, such as triangulation, the dual-LED method, and fingerprint database.

### 3.2. Coordinate System Transformation Relationship

**Figure 3:**
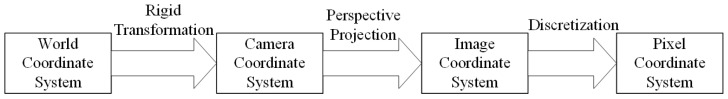
The visible light positioning system includes four coordinate systems.

We use the image sensor as the receiver of the visible light positioning system. Under ideal conditions, we can use the pinhole imaging model to describe the imaging principle of the image sensor. The pinhole imaging model can determine the position of the LED on the two-dimensional image. Four coordinate systems are involved in the imaging process: the world coordinate system, camera coordinate system, image coordinate system, and pixel coordinate system. The conversion relationship among them is shown in Figure 3.

First, we convert the LED coordinate from the world coordinate system to the camera coordinate system. The transformation process is a rigid body transformation. There is only rotation and translation without deformation. For example, the world coordinates of one LED are (X,Y,Z), and its coordinates in the camera coordinate system are $\left(X_{c},Y_{c},Z_{c}\right)$. The relationship between the two coordinates is ${\left[X_{c},Y_{c},Z_{c}\right]}^{T}=R{\left[X,Y,Z\right]}^{T}+T$, *T* is the offset in the coordinate system, and $R=R_{z}\left(\gamma \right)R_{y}\left(\beta \right)R_{x}(\alpha )$ is the rotation matrix, as follows:

(1) $$\begin{array}{cc} R= & \left[\begin{array}{ccc} \cos\gamma & -\sin\gamma & 0\\ \sin\gamma & \cos\gamma & 0\\ 0 & 0 & 1 \end{array}\right]\times \left[\begin{array}{ccc} \cos\beta & 0 & \sin\beta \\ 0 & 1 & 0\\ -\sin\beta & 0 & \cos\beta \end{array}\right]\times \left[\begin{array}{ccc} 1 & 0 & 0\\ 0 & \cos\alpha & -\sin\alpha \\ 0 & \sin\alpha & \cos\alpha \end{array}\right] \end{array}$$

where α, β, and γ are the three-dimensional rotation angles of the camera coordinate system relative to the world coordinate system. α represents the pitch angle, β represents the roll angle, and γ represents the azimuth angle.

Then, we convert $P(X_{c},Y_{c},Z_{c})$ in the camera coordinate system into (x,y) in the image coordinate system, according to the camera imaging principle, as follows:

(2) $$\begin{array}{c} s\left[\begin{array}{c} x\\ y\\ 1 \end{array}\right]=\left[\begin{array}{ccc} f & 0 & 0\\ 0 & f & 0\\ 0 & 0 & 1 \end{array}\right]\left[\begin{array}{c} X_{c}\\ Y_{c}\\ Z_{c} \end{array}\right] \end{array}$$

where *s* is the scale factor, s=a/r, *a* and *r* are the diameters of the LED in the image coordinate system and the world coordinate system, respectively. *f* is the focal length of the camera.

Finally, we convert LED coordinates in the image coordinate system to the pixel coordinate system. The difference between them lies in the origin coordinate and the unit of measurement.

## 4. Single LED Positioning Algorithm

In this section, we introduce our positioning algorithm.

### 4.1. Positioning Algorithm Overview

**Figure 4:**
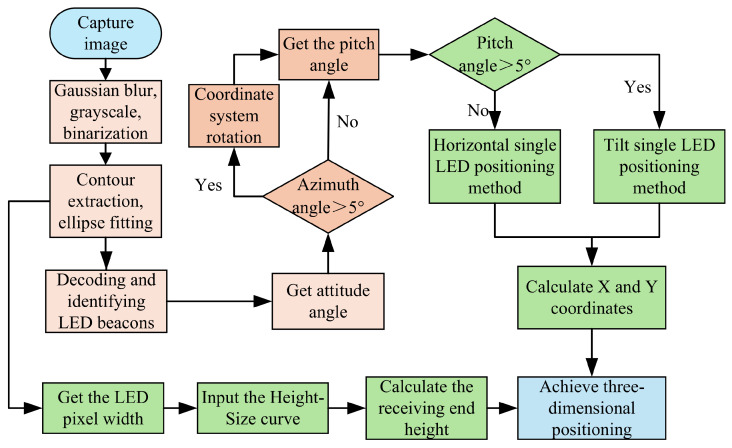
Three-dimensional positioning system under a single LED.

In actual application scenarios, if the number of LEDs in the camera field of view is less than 3, it cannot provide sufficient positioning information, resulting in positioning failure. Therefore, we design a single LED three-dimensional positioning algorithm. The algorithm calculates the horizontal and vertical coordinates and height of the receiver separately, avoiding the accumulation and transmission of errors in traditional positioning algorithms and improving the three-dimensional positioning accuracy. In order to cope with the tilted state of the receiver, we add an IMU to the receiver to obtain the receiver state and achieve positioning when the receiver is tilted. The system’s three-dimensional positioning is shown in Figure 4.

**Figure 5:**
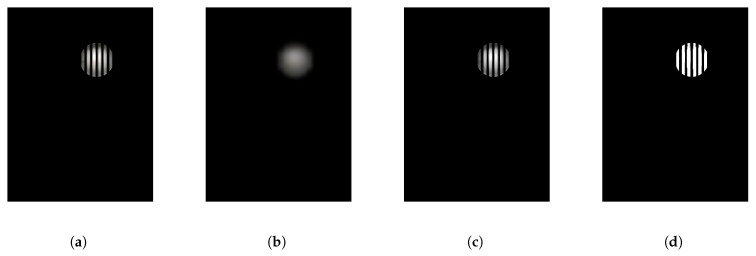
Image preprocessing results. (**a**) Original image. (**b**) Blurred image. (**c**) Grayscale image. (**d**) Binarized image.

After the transmitter transmits the beacon data, the image sensor in the receiver starts to capture the LED image. We preprocess the captured image, including Gaussian blur, grayscale, binarization, and other processes. The processed image is shown in Figure 5. We extract the LED outline from the preprocessed image, find the center of the centroid after ellipse fitting, and then decode the LED and identify its frequency.

In Figure 5d, we assume the image has m×n pixels, which means the image consists of m rows and *n* columns of pixels. The camera exposure time is *T*, the post-exposure processing time for each row is *d*, the LED frequency is *f*, and the width of each LED stripe generated in the image is *W*. We know as follows.

(3) $$\begin{array}{c} f=\frac{1}{2W\cdot \left(\frac{T}{m}+d\right)} \end{array}$$

After calculating the LED frequency, we must determine the receiver state to select different positioning algorithms.

First, we obtain the receiver’s azimuth and determine whether the camera coordinate system is parallel to the world coordinate system based on the value of the receiver’s azimuth angle. If the azimuth angle is greater than 5°, the receiver is considered to be in a non-parallel state. The camera coordinate system must be re-rotated to make it parallel to the world coordinate system. After rotating the coordinate system, the receiver can be in a parallel state; that is, the horizontal and vertical coordinates of the camera coordinate system are parallel to the world coordinate system.

Then, we determine whether the receiver is in a tilted state based on the pitch angle obtained by the IMU. If the pitch angle of the receiver is less than 5°, the receiver is considered to be placed horizontally. At this moment, the LED image is a perfect circle, and the center of the centroid in the image can be directly used to calculate the two-dimensional coordinates of the receiver. If the pitch angle is greater than 5°, the receiver is in a tilted state, and the LED image in the image is an ellipse. The image must be projected onto the horizontal plane to calculate the receiver’s two-dimensional coordinates.

Finally, to calculate the receiver’s height, we use the Gaussian blurred image of the LED to obtain the LED pixel width and input it into the height–size curve to obtain the corresponding receiver height. When both two-dimensional coordinates and height are obtained, the system achieves the three-dimensional visible light positioning.

### 4.2. Calculating Two-Dimensional Coordinate of Receiver

(1) Receiver horizontal placement.

**Figure 6:**
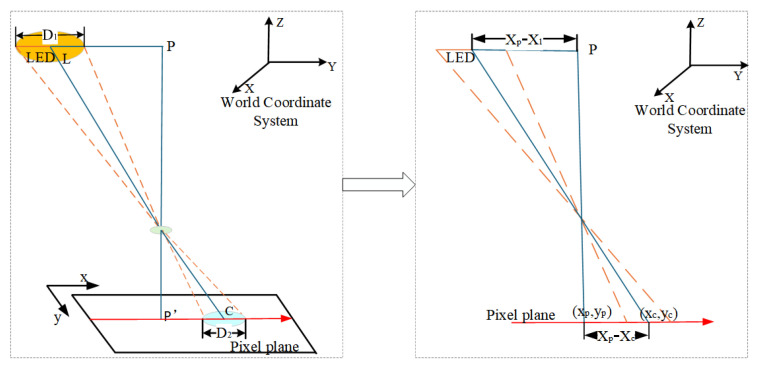
Receiver horizontal positioning principle.

When the receiver is placed horizontally, the calculation method of the *X* and *Y* coordinates of the receiver is shown in Figure 6. $L(X_{1},Y_{1})$ denotes the LED’s known position in the world coordinate system, and $P(X_{p},Y_{p})$ is the receiver’s unknown position in the world coordinates, $D_{1}$ and $D_{2}$ are the external dimensions of the LED and the diameter of the circular imaging area, respectively, and $C(X_{c},Y_{c})$ and $P^{\prime }\left(x_{p},y_{p}\right)$ are the central pixel coordinates of the LED and the image center coordinates, respectively. When the image coordinate system and the world coordinate system are parallel, according to the principle of similar triangles, as follows:

(4) $$\begin{array}{c} \frac{D_{1}}{D_{2}}=\frac{PL}{P^{\prime }C} \end{array}$$

further,

(5) $$\begin{array}{c} \frac{D_{1}}{D_{2}}=\frac{X_{p}-X_{1}}{x_{p}-x_{c}}=\frac{Y_{p}-Y_{1}}{y_{p}-y_{c}} \end{array}$$

we can calculate the receiver’s positioning as follows.

(6) $$\left\{\begin{array}{cc} X_{p} & =X_{1}+\left(x_{p}-x_{c}\right)\frac{D_{1}}{D_{2}}\\ Y_{p} & =Y_{1}+\left(y_{p}-y_{c}\right)\frac{D_{1}}{D_{2}} \end{array}\right.$$

(2) Receiver tilted placement.

**Figure 7:**
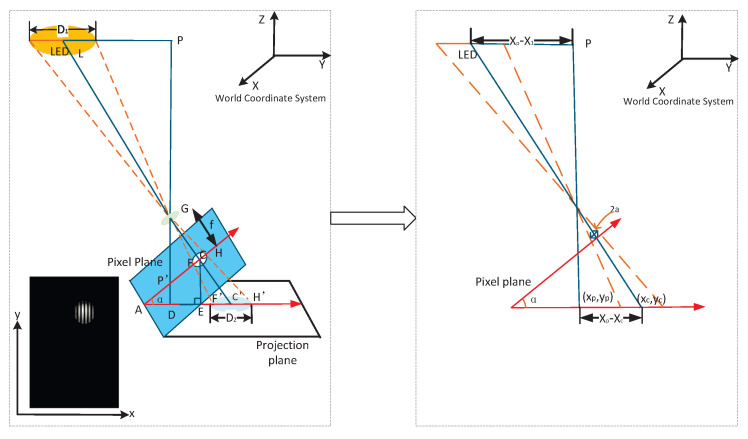
Receiver tilt positioning principle.

When the pitch angle of the receiver is greater than 5°, we consider the receiver to be tilted, and the positioning principle is shown in Figure 7. Due to the receiver’s tilt, the LED’s contour in the captured image is elliptical, affecting the positioning accuracy. We perform the ellipse fitting [29] on the LED image, find the centroid, and project the tilted pixel plane onto the horizontal plane. Then, we use the receiver horizontal placement method to calculate the receiver’s position.

In Figure 7, XOY is the world coordinate system of the positioning system. Point $L(X_{l},Y_{l})$ is the coordinate of the center of the LED in the world coordinate system. $D_{1}$ is the diameter of the LED. $C(x_{c},y_{c})$ is the coordinate of the center of the LED in the captured image, and $P^{\prime }\left(x_{p},y_{p}\right)$ is the center coordinate in the pixel plane. The coordinates of the receiver in the world coordinate system are $P(X_{p},Y_{p})$. Thus,

(7) $$\begin{array}{c} \frac{D_{1}}{F\prime H\prime }=\frac{PL}{DC\prime } \end{array}$$

(1) Solve EC′. In △GCH, tan∠GCH=f/a, where *a* is the semi-major axis of the elliptical LED imaging and *f* is the focal length of the camera. According to the angular relationship in Figure 7, the $∠ECC\prime =∠GCH-\left(90^{{}^{\circ}}-\alpha \right)$. Meanwhile, $∠ECC^{\prime }=arctan\left(EC^{\prime }/CE\right)$, and $EC^{\prime }$ is as follows,
  (8) $$EC^{\prime }=CE\cdot tan\left(arctan\frac{f}{a}-90^{{}^{\circ}}+\alpha \right)$$
(2) Solve $DC^{\prime }$. From Figure 7, we find that $DC^{\prime }=AE+EC^{\prime }-AD$. Let $\zeta =sin\alpha tan\left(arctanf/a-90^{{}^{\circ}}+\alpha \right)=EC^{\prime }/AC$. We get the following:
  (9) $$\begin{array}{c} \frac{PL}{DC^{\prime }}=\frac{X_{l}-X_{p}}{x_{c}\left(cos\alpha +\zeta \right)-x_{p}cos\alpha }=\frac{Y_{l}-Y_{p}}{y_{c}\left(cos\alpha +\zeta \right)-y_{p}cos\alpha } \end{array}$$
(3) Solve the positioning results $P(X_{p},Y_{p})$. Associating the above equations, the positioning coordinates are as follows:
  (10) $$\begin{array}{c} \left\{\begin{array}{c} X_{p}=X_{l}-\frac{D_{1}\left[x_{c}\left(cos\alpha +\zeta \right)-x_{p}cos\alpha \right]}{sx_{c}\zeta }\\ Y_{p}=Y_{l}-\frac{D_{1}\left[y_{c}\left(cos\alpha +\zeta \right)-y_{p}cos\alpha \right]}{2x_{c}\zeta } \end{array}\right. \end{array}$$
  Until now, we have found the *X* and *Y* coordinates of the receiver, achieving the high-precision two-dimensional positioning.

### 4.3. Calculating the Receiver’s Height

**Figure 8:**
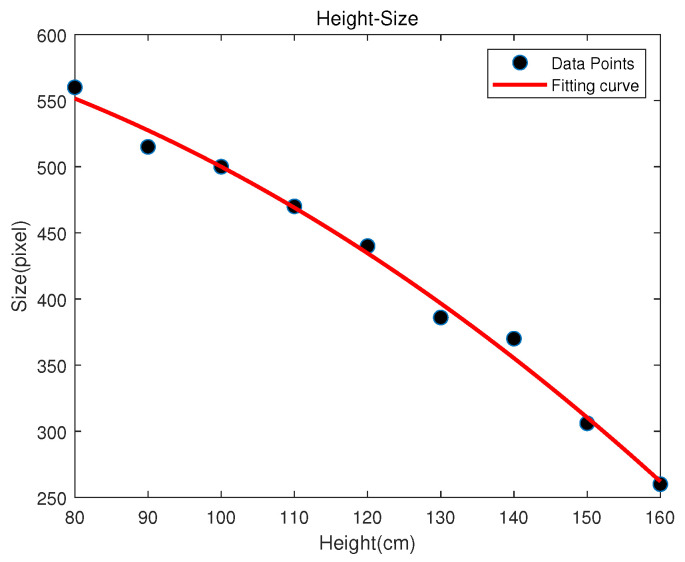
Height–size fitting curve.

In order to obtain the *Z* coordinate of the receiver, we use a height calculation algorithm through the second-order polynomial fitting method. We first use the second-order polynomial fitting curve to establish a linear relationship between the LED width in the image and the height collected by the database. Specifically, when the receiver is not tilted, we collect the LED images at different heights to establish the discrete height–size database. The database stores the LED’s pixel width and corresponding receiver height in the world coordinate system. These discrete data are fitted with a second-order polynomial to obtain a continuous height–size curve, and a mapping function relationship between the LED pixel width and the vertical distance from the LED to the receiver is established, as shown in Figure 8.

In Figure 8, the red represents the fitting curve, and the black point represents the collected discrete data. The fitted curve expression is $Size=-0.017{Height}^{2}+0.55Height+618.65$. It can express the linear relationship between the receiver height and LED pixel width.

(1) When the receiver is placed horizontally, the pixel width of the LED in the image is input into the curve, and the corresponding height is obtained. Since the height of our experimental site is 2.65 m, the *Z* coordinate of the receiver can be expressed as follows.
  (11) $$\begin{array}{c} Z=265-Height \end{array}$$
(2) When the receiver is tilted, the image is projected onto a plane parallel to the ground, as shown in Figure 7. F′H′ is approximately the diameter of the LED in the image carried in a non-tilted state, which is obtained according to Equation (7). Substitute F′H′ into the fitting curve to calculate the height of the receiver.

## 5. Dynamic Positioning Algorithm Through FA-UPF

In this section, we focus on the dynamic positioning algorithm with a firefly-assisted unscented particle filter.

### 5.1. Unscented Particle Filter Analysis

For the case where the system has no light source, most visible light positioning systems use the Kalman filter [30] to fuse visible light positioning with IMU data for achieving the positioning. However, the Kalman filter uses a linear approximation to calculate the Jacobian matrix nonlinearly and requires a manual initial position setting. Therefore, we use the unscented particle filter [31], which combines the advantages of the Kalman filter and the particle filter.

The unscented particle filter combines the nonlinear processing capability of the unscented transform with the particle filter’s strong robustness, enabling better state estimation capabilities in complex nonlinear systems. It still has shortcomings in dynamic visible light positioning without light sources. There are two reasons: (1) IMU’s noise and drift error grow rapidly over time. Without light sources for a long time, the prediction results of the unscented particle filter will deviate from the actual trajectory. (2) In the absence of light sources, due to insufficient observations, the particle degradation phenomenon of unscented particle filters is more serious, resulting in a decrease in estimation accuracy, and the computational cost increases significantly with the increase in the number of particles, which is not suitable for visible light positioning systems with high real-time requirements.

To compensate for the dynamic positioning error from the unscented particle filter in the absence of light sources, we use the firefly algorithm (FA) to assist the unscented particle filter in performing the receiver positioning. The firefly-assisted unscented particle filter algorithm can dynamically adjust the particle sampling distribution through FA and optimize the resampling of the unscented particle filter, improving the prediction accuracy. In addition, the error correction mechanism from FA can also effectively reduce the impact of IMU cumulative errors on unscented particle filter prediction results.

### 5.2. Firefly-Assisted Unscented Particle Filter for Dynamic Positioning

In the later step of the unscented particle filter, the problem of particle depletion leads to reduced positioning accuracy. We introduce the firefly algorithm [32] to optimize and improve the unscented particle filter.

The position update of the firefly algorithm requires each firefly to interact with all other fireflies at the current moment. If this algorithm is applied to unscented particle filter positioning, the calculation time will significantly increase, seriously affecting the real-time positioning performance. Therefore, the firefly algorithm needs to be appropriately improved.

(1) Update the firefly brightness.

In the unscented particle filter algorithm, particles with larger weights are closer to the actual value. In the firefly algorithm, the brighter the firefly, the better its position in space. Therefore, each particle can be regarded as an individual firefly. The calculated particle weight represents the firefly’s brightness as follows:

(12) $$I=w_{k}^{i}$$

(2) Improve the firefly attraction.

In the standard firefly algorithm, each firefly is attracted by adjacent fireflies to update its position. The farther away the fireflies are, the smaller their relative attraction and role in guiding position updates. We design each particle to update its position only by comparing it with the global optimal particle at the current moment. Suppose the distance from the global optimal value is too large. In that case, the degree of attraction will be minimal, causing the particles to be unable to move to the high-likelihood area or move slowly. So the attraction is improved as follows:

(13) $$\beta =\left(\beta_{\max}-\beta_{\min}\right)\times \exp\left(-\gamma r_{ij}^{2}\right)+\beta_{\min}$$

where $\beta_{\max}$ is the maximum attraction and $\beta_{\min}$ is the minimum attraction.

The improved method still satisfies the requirement that the closer the distance between particles, the greater the relative attraction. At the same time, particles far from the global optimal value can be guided by the minimum attraction to update their positions.

(3) Improve the firefly position updating.

In the standard firefly algorithm, each firefly needs to calculate the distance to all other fireflies and update its position interactively with them, respectively. Suppose it is directly substituted into the particle filter. In that case, it will greatly increase the complexity of the calculation and the position estimation time, making it difficult to meet the positioning requirements. Therefore, the particles at each moment are only moved closer to the particles at the global optimal position to reduce the algorithm calculation time. The improvement is as follows:

(14) $$x_{m}^{i}\left(k\right)=x_{m-1}^{i}\left(k\right)+\beta \times \left(xbest_{m-1}\left(k\right)-x_{m-1}^{i}\left(k\right)\right)+\alpha \times \left(rand-0.5\right)$$

where $x_{m}^{i}\left(k\right)$ is the state of particle *i* at time *k* after the m iteration and $x_{m-1}^{i}\left(k\right)$ is the state of particle *i* at time *k* after the m−1 iteration. $xbest_{m-1}\left(k\right)$ is the particle state with the largest weight among all particles after the m−1 iteration at time *k*.

## 6. Evaluation

In this section, we briefly introduce the system components and verify the performance of the localization system. In the paper, all positioning results shown in the figures are calculated and presented in the world coordinate system.

### 6.1. Platform of Hardware

The system’s transmitter and receiver model is shown in Table 1, and the frequency allocation is shown in Table 2. We modulate the LED to different frequencies to give different beacon information. In the positioning process, the world coordinates of the LED can be obtained according to the calculated LED frequency for positioning.

**Figure 9:**
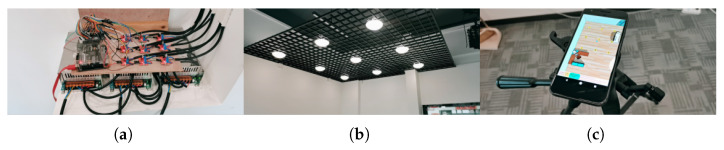
Scenario of the experiment. (**a**) Transmitter. (**b**) Ceiling layout. (**c**) Smartphone receiver.

Figure 9 shows the experimental test scenario. Figure 9a shows the LED source at the transmitter, and Figure 9b shows the ceiling LED layout. Figure 9c shows the receiver of the smartphone. In the system, the software system takes 350 ms to perform positioning, with 150 ms for capturing and storing the image, 150 ms for performing the image processing, and 20–30 ms for the positioning algorithm. There is a time redundancy gap between the steps for removing the effect of possible errors and bugs in the software code.

### 6.2. Positioning Error

**Figure 10:**
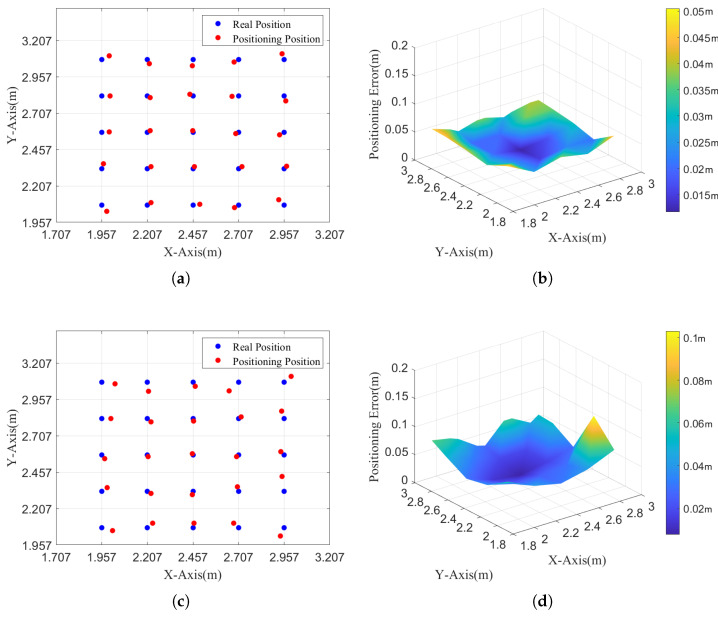
The positioning result and error under different tilt angles. (**a**) 2D positioning results under 0° tilt angle. (**b**) 3D positioning errors under 0° tilt angle. (**c**) 2D positioning results under 10° tilt angle. (**d**) 3D positioning errors under 10° tilt angle.

We measure the system’s static positioning error for a receiver at different tilt states. 5×5 points are selected within the 2 m × 2m area in the experiment. Under our experimental setup, the effective horizontal positioning range of a single LED is approximately 0.5 m × 0.5 m, within which the positioning error remains below 10 cm in most cases. We perform tests at tilt angles of $0^{{}^{\circ}}$ and $10^{{}^{\circ}}$, respectively. The positioning results under the tilt angles of $0^{{}^{\circ}}$ and $10^{{}^{\circ}}$ are shown in Figure 10.

Figure 10a,c show the two-dimensional positioning results and three-dimensional positioning errors of the single-LED positioning algorithm under the tilt angle of 0°. The average positioning error is 3.08 cm. The minimum positioning error is 1.17 cm, and the maximum positioning error is 5.05 cm.

Figure 10b,d show the two-dimensional positioning results and three-dimensional positioning errors of the single-LED positioning optimization algorithm at a tilt angle of 10°. The average positioning error is 4.13 cm, which is 34% higher than that at a tilt angle of 0°. The minimum positioning error is 0.76 cm, and the maximum positioning error is 10.3 cm.

**Figure 11:**
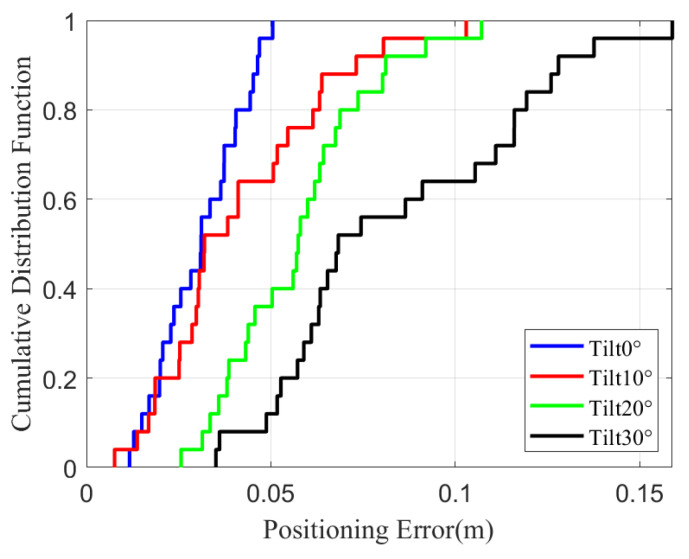
Cumulative distribution function of positioning error at different tilt angles.

In Figure 11, we show the cumulative distribution function (CDF) of positioning error at different tilt angles. From Figure 11, we can find that the more parallel the receiver surface is to the horizontal, the smaller the system positioning error is. It is because the smaller the smartphone’s tilt angle, the smaller the deformation of the captured LED pixels, so more accurate image processing and positioning accuracy are achieved. Meanwhile, the average positioning error of the system is 5.34 cm, and 60% of positioning errors are within 10 cm, which means high-precision design requirements are completed.

### 6.3. Dynamic Trajectory Analysis

In this section, we analyze the system’s dynamic positioning performance from two perspectives: (1) path-wise tracking accuracy; (2) the statistical distribution of dynamic positioning errors.

#### 6.3.1. Path-Wise Tracking Accuracy

(1) Dynamic positioning error under the single LED.

**Figure 12:**
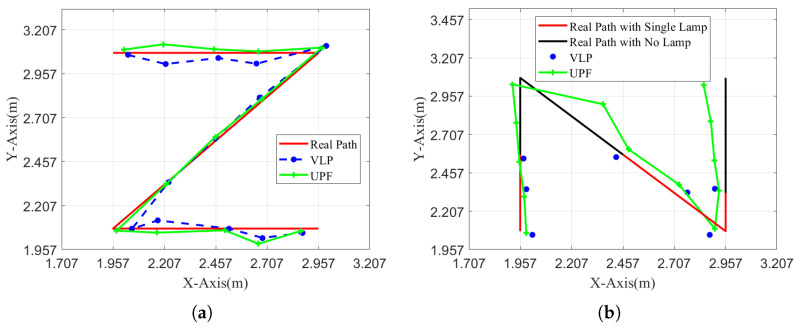
Dynamic positioning performance based on FA-UPF and VLP under different paths. (**a**) The Z path. (**b**) The N path.

In Figure 12a, we show the system’s positioning accuracy with the FA-UPF under the single LED. During the experiment, we select a *Z* path within the 2 m × 2 m area and let the smartphone move along the path *Z*. The red line is the actual path of the smartphone. The blue and green lines are drawn according to the positioning results, respectively, without FA-UPF and with FA-UPF. From Figure 12a, we can find that the green path is closer to the red path, which means the FA-UPF brings the system higher positioning precision.

(2) Dynamic positioning errors under the occasional no-LED.

Figure 12b shows the system’s positioning accuracy with FA-UPF in the occasional no-LED. During the experiment, we select an *N* path within the 2×2 area and temporarily turn off some LEDs to simulate the situations without LEDs. We record the results of dynamic positioning with FA-UPF. The red path represents the actual path of the smartphone’s movement when only one LED in FoV exists. The black path represents the actual path of the smartphone’s movement in a no-LED situation. From Figure 12b, we can find that the visible light positioning system is disabled immediately when there is no LED in FoV, but the FA-UPF can predict the current position according to the previous state and inertia data to provide positioning service.

#### 6.3.2. Error Distribution in Dynamic Scenarios

(1) Histogram and CDF of positioning error for Z path.

**Figure 13:**
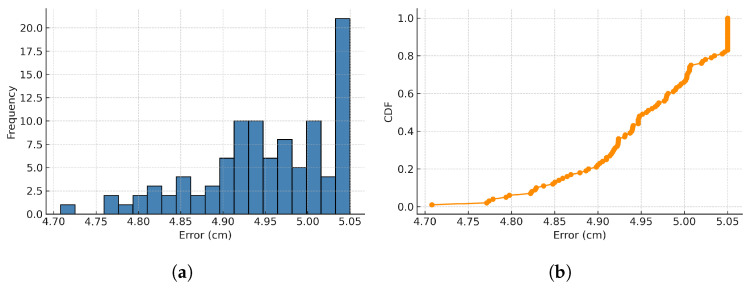
Z path positioning error distribution. (**a**) Positioning error histogram. (**b**) Cumulative distribution function.

Figure 13 shows the histogram and cumulative distribution function (CDF) of the positioning error during Z-path movement. When the LED was turned on, the errors have a mean of approximately 4.97 cm, a minimum of 1.17 cm, and a maximum of 5.05 cm. The histogram shows that most of the positioning errors are concentrated around 4.9 cm to 5.0 cm, while the CDF curve rises steeply, with 90% of errors falling below 5.05 cm. This confirms that, in dynamic conditions, the system maintains centimeter-level accuracy under an LED turned on stably.

(2) Histogram and CDF of positioning error for N path.

**Figure 14:**
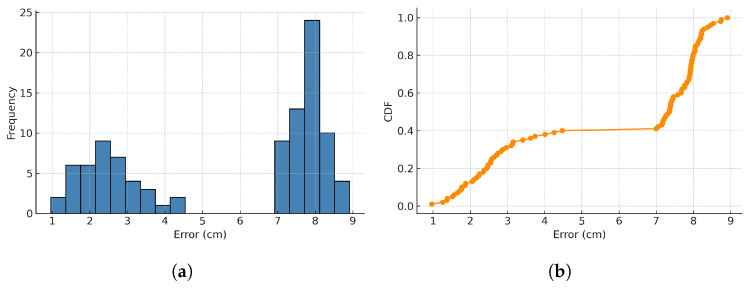
N path positioning error distribution. (**a**) Positioning error histogram. (**b**) Cumulative distribution function.

Figure 14 presents the histogram and CDF of the positioning error under the N path, where some LEDs are intentionally turned off to simulate periods of LED absence. The error distribution exhibits two clusters: one in the low-error region (0.8–3 cm) corresponding to normal LED detection, and another in the high-error region (7–9 cm) when LED signals are unavailable. Although the errors increase during LED outages, the FA-UPF algorithm effectively constrains the error growth by relying on IMU prediction. The system still achieves acceptable accuracy when partially LED signals are turned off.

Further, our data analysis reveals that when the smartphone suddenly changes the direction of movement and there is no LED in the FoV of the receiver, it leads to a significant error between the predicted position and the actual position after the change of direction. The angle of the FA-UPF can only be obtained from the IMU, and the prediction effect is significantly reduced. The average positioning error with FA-UPF in path *N* is 6.45 cm, and the average error of the no-LED part of the path is 8.91 cm. The system can keep centimeter-level high-precision positioning in the occasional no-LED situation.

While our experiments include two representative motion trajectories (Z and N), the system is expected to maintain robust performance across other types of trajectories due to the FA-UPF’s ability to compensate for temporary LED absence and motion uncertainty. However, sharp turns or irregular motion may lead to slightly higher prediction errors, especially when LED signals are unavailable. Future work will include more complex and realistic movement paths to evaluate the generalization of the proposed approach.

### 6.4. Discussion on Ambient Light Robustness

In practical environments, visible light positioning systems must operate reliably under various ambient lighting conditions. Our system is designed to be robust to both natural light (e.g., sunlight) and artificial light sources (e.g., ceiling lamps), as the LED transmitters are frequency-modulated and uniquely identifiable through image-based frequency decoding. Furthermore, Gaussian blurring and binarization preprocessing techniques help suppress background illumination and enhance LED signal isolation. Nevertheless, in extremely bright environments, overexposure may hinder LED detection. In such cases, adaptive exposure control and optical filters (e.g., bandpass filters) could be employed to improve robustness.

### 6.5. Impact of LED Frequency on Positioning Performance

The proposed system relies on frequency-modulated LED signals to identify and distinguish different beacons. The driving frequency of the LEDs determines the width of the LED stripes captured in the image sensor. In our system, the frequency is calculated based on the number and width of these stripes. Therefore, significant changes in driving frequency may affect stripe clarity and the accuracy of frequency decoding, especially if the frequency is too high or too low relative to the camera’s exposure settings. However, within a reasonable operating range (e.g., 600 Hz to 2.2 kHz in our experiments), the decoding algorithm maintains stable performance. This demonstrates that the system is robust to moderate changes in LED driving frequencies. For extreme cases, adjusting the camera’s exposure time or employing adaptive frequency calibration may be necessary.

### 6.6. Comparative Analysis with Related Works

To evaluate the effectiveness of the proposed system, we compare it with several representative image sensor-based VLP methods from recent literature. Table 3 summarizes the comparison in terms of LED requirements, dimensionality, need for angle sensors, and robustness to LED absence. Unlike prior works [13,18], which require multiple LEDs or fail under sparse lighting, our method achieves 3D positioning using only a single LED and maintains accurate tracking when the LED is not visible by leveraging the FA-UPF algorithm. Moreover, the system does not require angle sensors, which reduces hardware cost and complexity. These advancements demonstrate that our method extends the applicability of VLP systems in constrained or dynamic environments.

## 7. Conclusions

In this paper, we have solved typical issues in visible light positioning systems, such as sparse LED layouts, IMU drift errors, and the inability to locate in the absence of light sources. The proposed system achieves an average static positioning error of 5.34 cm within a 2 m × 2 m × 2.65 m area. Under a 10° tilt angle, the error remains within 10.3 cm. In addition, to enhance the system’s performance, we have proposed a single-LED 3D visible light positioning algorithm and a firefly-assisted unscented particle filter algorithm. The FA-UPF algorithm enables position prediction in no-LED scenarios with an average error of 6.45 cm and a worst-case error of 8.91 cm. Over 70% of the test points yield errors below 10 cm, demonstrating the system’s high accuracy and robustness. Experimental results have demonstrated that the proposed algorithms can achieve centimeter-level indoor positioning accuracy under various environments.

Despite the promising results, the proposed system has several limitations. First, although the system achieves 3D positioning with a single LED, the accuracy may degrade under strong ambient light or when the LED image is significantly occluded. Second, the FA-UPF algorithm depends on the IMU data for prediction in no-light conditions, and its performance may decline under long-term drift or sudden acceleration. Finally, the current implementation is tested in controlled indoor environments, and generalization to larger, dynamic spaces remains unverified.

To address these limitations and further improve system performance, we plan to pursue the following directions in future work:

(1) Multi-sensor fusion: Integrating UWB or WiFi to compensate for IMU drift in prolonged LED absence scenarios.
(2) Dynamic environmental adaptation: Developing real-time calibration for ambient light interference and reflective surfaces (e.g., mirrors).
(3) Large-scale deployment: Extending the algorithm to multi-floor 3D positioning with sparse LED layouts.
(4) Theoretical analysis: Modeling the impact of LED driving frequency fluctuations on positioning robustness under mobile camera conditions.

## Figures and Tables

**Table 1:** Hardware model.

Parameters	Value
Experimental area size	2.0 × 2.0 × 2.65 m^3^
LED Model	CXA1512 (Cree Inc., Durham, NC, USA)
LED Maximum Power	21.6 W
Lampshade Diameter	20 cm
Chip type	Cyclone IV EP4CE6F (Intel/Altera Corp., San Jose, CA, USA)
Amplifier circuit	LM324N (Texas Instruments Inc., Dallas, TX, USA)
MOS type	IRF520 (Infineon Technologies AG, Neubiberg, Germany)
Power	GPS-430C (GW Instek, New Taipei City, Taiwan)
Smartphone Model	Google Pixel (Google LLC, Mountain View, CA, USA)
Screen resolution	1920 × 1080
System version	Android 7.0 / 8.0 (Google LLC, Mountain View, CA, USA)
Front camera resolution	2448 × 3264
Camera exposure time	100 μs
Camera sensitivity	64

**Table 2:** LED frequency allocation.

LED label	Frequency	LED Label	Frequency
A	600 Hz	B	800 Hz
C	1 kHz	D	1.2 kHz
E	1.4 kHz	F	1.6 kHz
G	1.8 kHz	H	2 kHz
I	2.2 kHz	…	…

**Table 3:** Comparison with representative image sensor-based VLP methods.

Method	LED Number	3D Positioning	Robust to LED Absence	Error
Li et al. [12]	≥3	No	No	12 cm
Mao et al. [18]	1	No	No	1.7 cm
Hao et al. [19]	1	Yes	No	10 cm
This work	1	Yes	Yes (FA-UPF)	5.34 cm (LED on)
				6.45 cm (No LED)

## Data Availability

The original contributions presented in this study are included in the article. Further inquiries can be directed to the corresponding author.
